# Interaction between serum cotinine and body mass index on asthma in the children: a cross-sectional study

**DOI:** 10.1186/s12887-022-03571-0

**Published:** 2022-08-23

**Authors:** Li He, Xiaojing Xi

**Affiliations:** 1 School of Medicine, Xinjiang University of Science & Technology, No.89, Beijing Road, Yingxia Township, 841000, Korla City, Xinjiang, People’s Republic of China; 2 Department of Quality Management, Medical Department, The First Affiliated Hospital of Xinjiang Medical University, Urumqi, 830054 Xinjiang, People’s Republic of China

**Keywords:** Asthma, Serum cotinine, BMI z-score, Children

## Abstract

**Background:**

The purpose of this study was to explore the interaction between serum cotinine (a marker of environmental tobacco smoke exposure) and body mass index (BMI) on asthma in children.

**Methods:**

This cross-sectional study relied on representative samples of American children included in the National Health and Nutrition Examination Survey in 1999–2018. Multivariate logistic regression analyses were to evaluate the association between serum cotinine level, BMI z-score and asthma. Serum cotinine was dichotomized at 0.0436 ng/mL. Interactions were examined by the estimated joint effect of BMI and serum cotinine levels. We also performed interaction analyses in age and ethnicity subgroups.

**Results:**

Among the 11,504 children aged 3 to 12 years included in the analysis, 15.86% (*n* = 1852) had childhood asthma, 15.68% (*n* = 1837) were overweight, and 17.31% (*n* = 2258) were obese. Compared to low serum cotinine, high serum cotinine was significantly associated with asthma [odds ratio (OR) = 1.190, 95% confidence interval (CI): 1.004–1.410]. Overweight (OR = 1.275, 95%CI: 1.079–1.506) and obesity (OR = 1.636, 95%CI: 1.354–1.977) were significantly associated with asthma compared with normal weight. The adjusted attributable proportion of interaction = 0.206 (95%CI: 0.075–0.337) and the adjusted synergy index = 1.617 (95%CI: 1.126–2.098) indicated that there was a significant synergistic effect of serum cotinine levels and BMI on asthma. In males, females, non-Hispanic White and other Hispanic, there were synergistic interactions between serum cotinine levels and BMI on asthma.

**Conclusion:**

A synergistic interaction between serum cotinine and overweight/obesity on childhood asthma was found. For children with asthma, both intensive weight interventions in overweight or obese children and intensive passive smoking interventions in children exposed to the environment may be important.

**Supplementary Information:**

The online version contains supplementary material available at 10.1186/s12887-022-03571-0.

## Background

Asthma is the most common chronic disease in childhood with high morbidity and associated medical costs [1, 2]. Asthma is a respiratory disease that causes repeated coughing, shortness of breath and wheezing [1, 3]. In the United States, 1 in 12 children between the ages of 0 and 17 have asthma [1].

Children exposed to cigarette smoke (environmental tobacco smoke) are at greater risk of lung disease, respiratory infections, and asthma attacks [4]. Parental smoking is a common source of children's exposure to cigarettes [5, 6]. Children who live in homes with two or more smokers were about three times more likely to be exposed to second-hand smoke than those who do not live with smokers [7]. Serum cotinine levels can reflect the passive exposure of children to cigarettes, which is the main metabolite of nicotine with a long half-life, about 15–20 h [8, 9]. Some epidemiological studies found that serum cotinine levels were associated with the incidence and severity of childhood asthma [10, 11].

Obesity is now considered to be the relevant risk factor for childhood asthma, elevated body mass index (BMI) (overweight or obesity) is a common complication of asthma [12], and studies have shown that obesity or weight gain usually precedes an asthma attack [12, 13]. In addition, a study based on the data from the United States National Health and Nutrition Examination Surveys (NHANES) 1999–2012 showed that BMI increased the risk of children's active and passive exposure to tobacco smoke were assessed using the optimal biomarker serum cotinine [14]. Serum cotinine and overweight/obesity are known to be associated with childhood asthma. Two risk combinations may develop separately when passive smoking co-occurs with overweight/obesity, however, there was limited research on the interaction between serum cotinine and BMI on childhood asthma.

Therefore, the study was to explore the interaction between serum cotinine and BMI on asthma in general US children.

## Methods

### Study population

**Fig. 1:**
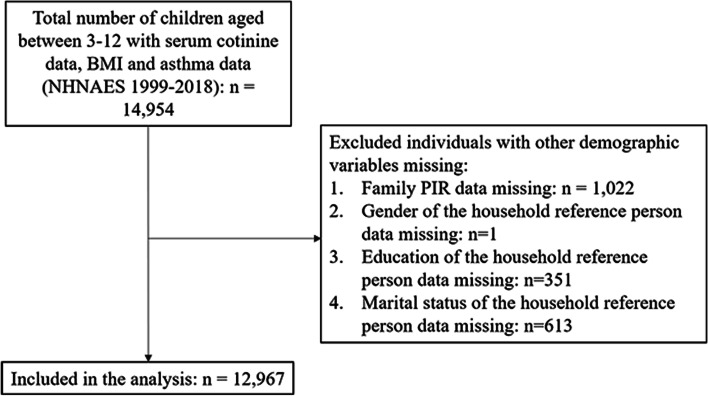
Flowchart of the systematic selection process

Data of this cross-sectional study were retrieved from nine surveys [1999–2000, 2001–2002, 2003–2004, 2005–2006, 2007–2008, 2009–2010, 2011–2012, 2013–2014, 2015–2016, 2017–2018] United States National Health and Nutrition Examination Surveys (NHANES). NHANES are multifaceted cross-sectional sampling designs administered to a representative sample of civilian non-institutionalized individuals within the U.S. population. NHANES includes interviews, physical examinations and laboratory assessments. The database enrolled 14,954 children aged 3 to 12 years with serum cotinine, BMI and asthma data from 1999–2018. Participants were excluded if the following data were missing: (1) ratio of family income to poverty (PIR) (*n* = 1022); (2) household reference person’s gender (*n* = 1); (3) household reference person’s education (*n* = 351); and (4) household reference person’s marital status (*n* = 613). The household reference person is the first household member, 18 years of age or older who is listed on the Screener household member roster who owns or rents the residence where members of the household reside. We identified 11,504 children in the final analytic sample for our primary analysis. The flow chart of the systematic selection process is shown in Fig. 1. The National Center for Health Statistics (NCHS) Ethics Review Committee granted ethics approval. All individuals provided written informed consent before participating in the study.

### Asthma diagnosis

The diagnosis of ever asthma was determined by the question “has a doctor or other health professional ever told you that you have asthma?”. If the answer to the question was yes, the child was considered to have asthma. Children with current asthma were identified by responding affirmatively to the question “During the past 12 months, have you had an episode of asthma or an asthma attack?”.

### Explanatory variables

The serum cotinine level was determined from the venous blood samples of the study participants. The method of an isotope dilution–high-performance liquid chromatography / atmospheric pressure chemical ionization tandem mass spectrometry (ID HPLC-APCI MS/MS) was used and serum cotinine was reported in ng/L. This method was used in all the assessments from 1999 to 2018. A detailed description of serum cotinine measurement in NHANES is available online (https://wwwn.cdc.gov/nchs/data/nhanes/2017-2018/labmethods/COT-J-MET-508.pdf). In this analysis, we used a serum cotinine cutoff of 0.0436 ng/mL (median) to define serum cotinine levels, with < 0.0436 ng/mL as low-level serum cotinine and ≥ 0.0436 ng/mL as high-level serum cotinine.

The sex-specific BMI (body mass index)-for-age reference of Centers for Disease Control and Prevention (CDC) and LMS method were used to calculate the BMI z-score [15]. Subjects were categorized based on BMI percentiles as follows: Group A [underweight and normal weight (< 85th percentile)], Group B [overweight (≥ 85th percentile to < 95th percentile)], and Group C [obesity (≥ 95th percentile)] [15], which was consistent with other epidemiological studies [14, 16].

### Covariates

The covariates contained some demographic characteristics age, gender, ethnicity (Mexican American/ other Hispanic/ non-Hispanic white/ non-Hispanic black/ other races). The household reference person’s gender, age, education [less than 9th grade/ 9-11th grade/ high school graduate/ some college or associates (AA) degree/ college graduate or higher], and marital status (divorced/ living with partner/ married/ never married/ separated/ widowed). Data on whether anyone smoked in home was also collected. The ratio of family income to poverty (PIR) was calculated by dividing household income by poverty guidelines, specific to the appropriate year and the state of the participant [17]. Household income was reported as a range value, the midpoint of the range was used to calculate the PIR. The lower the PIR, the higher the poverty level. The PIR was 1.0 (income at 100% of poverty level) indicated that the person was on the poverty line; the ratio was 0.5 (income at 50% of poverty level) meant that the person's family income was half of the poverty line.

### Statistical analysis

SDMVSTRA (masked variance unit pseudo-stratum variable for variance estimation) was used for stratification, SDMVPSU (masked variance unit pseudo-PSU variable for variance estimation) was used for variance estimation, and WTMEC2YR (the two-year sample weighed) were used to weight calculation. All measured variables were expressed as counts and weighted percentages [n (weighted %)] or mean and standard error (SE). Children with and without asthma were compared using weighted t-tests and Chi-square tests. First, the frequencies of children with and without asthma were calculated and a univariate analysis was performed. Whether there were significant differences between participants who were included and those who were excluded from the analysis was examined. Second, multivariable logistic regression analyses were conducted to evaluate the association between serum cotinine level, BMI and asthma. Three multivariate logistic regression models were built. Model 1 was the crude model which did not adjust any confounders, age and gender were adjusted in Model 2, and age, gender, ethnicity, gender of the household reference person, and marital status of the household reference person were adjusted in Model 3. Third, interaction analyses were conducted. The estimated joint effect of BMI and serum cotinine levels was greater than the sum of the independent effect of the two factors, indicating the additive interaction between BMI and serum cotinine levels in association with asthma [18]. There was no additive interaction when the confidence interval of the attributable proportion of interaction contained 0 and the synergy index contained 1. For those with current asthma, sensitivity analysis was performed to explore the interaction between BMI and serum cotinine on current asthma. In addition, interaction analyses in age and ethnicity subgroups were also performed. The software of SAS version 9.4 (SAS Institute, Inc, Cary, NC) was used for all analyses. All statistical tests used two-sided tests and *P* < 0.05 was considered statistically significant.

## Results

### Characteristics of the study population

A total of 12,967 children aged 3 to 12 years with serum cotinine levels from NHANES were included in the analysis (Fig. 1). The mean (SE) of age was 7.87 (0.03). 6,389 (47.90%) were females, and 6,578 (52.10%) were males. Overall, 16.01% (*n* = 2076) of children had asthma, 15.59% (*n* = 2056) of children were overweight, and 17.08% (*n* = 2506) were obese (Table 1).

**Table 1:** Basic information and comparison between children with and without asthma

Characteristic	Total (*n* = 12,967)	Whether had asthma		Statistic	*P*
		Yes (*n* = 2,076)	No (*n* = 10,891)		
Age, years, Mean (SE)	7.87 (0.03)	8.32 (0.08)	7.79 (0.04)	t = 5.90	< 0.001
PIR, Mean (SE)	2.35 (0.04)	2.26 (0.06)	2.36 (0.04)	t = -1.80	0.074
Serum cotinine, ng/ml, n (%)^1^				χ^2^ = 12.434	< 0.001
Low-level (< 0.0436)	6,182 (49.86)	845 (44.98)	5,337 (50.75)		
High-level (≥ 0.0436)	6,785 (50.14)	1,231 (55.02)	5,554 (49.25)		
Gender, n (%)^1^				χ^2^ = 32.192	< 0.001
Male	6,578 (52.10)	1,220 (59.63)	5,358 (50.73)		
Female	6,389 (47.90)	856 (40.37)	5,533 (49.27)		
Ethnicity, n (%)^1^				χ^2^ = 62.465	< 0.001
Mexican American	3,537 (15.24)	414 (11.63)	3,123 (15.90)		
Other Hispanic	1,087 (7.04)	223 (8.81)	864 (6.72)		
Non-Hispanic White	3,674 (55.86)	528 (52.42)	3,146 (56.49)		
Non-Hispanic Black	3,466 (13.91)	727 (19.27)	2,739 (12.93)		
Other races	1,203 (7.94)	184 (7.88)	1,019 (7.96)		
BMI groups, n (%)^1^				χ^2^ = 42.440	< 0.001
Croup A	8,405 (67.33)	1,202 (59.17)	7,203 (68.81)		
Croup B	2,056 (15.59)	342 (17.17)	1,714 (15.30)		
Croup C	2,506 (17.08)	532 (23.65)	1,974 (15.89)		
Gender of the household reference person^2^, n (%)^1^				χ^2^ = 12.851	< 0.001
Male	5,895 (50.37)	794 (45.42)	5,101 (51.26)		
Female	7,072 (49.63)	1,282 (54.58)	5,790 (48.74)		
Age of the household reference person, years, Mean (S.E)	34.96 (0.22)	35.16 (0.53)	34.93 (0.23)	t = 0.43	0.669
Education of the household reference person, n (%)^1^				χ^2^ = 8.893	0.064
Less than 9th grade	1,624 (8.36)	193 (7.08)	1,431 (8.59)		
9-11th grade	2,849 (17.97)	482 (19.66)	2,367 (17.66)		
High school graduate	3,079 (24.22)	502 (24.48)	2,577 (24.17)		
Some college or AA degree	3,291 (26.53)	577 (28.24)	2,714 (26.22)		
College graduate or higher	2,124 (22.92)	322 (20.53)	1,802 (23.35)		
Marital status of the household reference person, n (%)^1^				χ^2^ = 30.438	< 0.001
Divorced	1,252 (9.49)	240 (11.05)	1,012 (9.21)		
Living with partner	888 (5.61)	141 (6.27)	747 (5.49)		
Married	8,088 (70.08)	1,140 (63.68)	6,948 (71.24)		
Never married	1,646 (7.97)	343 (10.85)	1,303 (7.45)		
Separated	706 (4.13)	132 (4.72)	574 (4.02)		
Widowed	387 (2.73)	80 (3.44)	307 (2.60)		
Anyone smokes in home, n (%)^1^				χ^2^ = 2.677	0.102
No	10,235 (78.59)	1,582 (76.57)	8,653 (78.96)		
Yes	2,732 (21.41)	494 (23.43)	2,238 (21.04)		

PIR, ratio of family income to poverty; *BMI* Body mass index, *SE* Standard error, Group A: underweight and normal weight (< 85th percentile); Group B: overweight (≥ 85th percentile to < 95th percentile); and Group C: obesity (≥ 95th percentile)

^1^ weighted %

^2^ The household reference person is the first household member, 18 years of age or older who is listed on the Screener household member roster who owns or rents the residence where members of the household reside

### Comparison of children with asthma and without asthma

Table 1 shows the significant differences in age (*P* < 0.001), serum cotinine (*P* < 0.001), BMI groups (*P* < 0.001), gender (*P* < 0.001), ethnicity (*P* < 0.001), gender of the household reference person (*P* < 0.001), and marital status of the household reference person (*P* < 0.001) between children with asthma and without asthma. However, there were no significant differences in PIR, age of the household reference person, education of the household reference person and anyone smokes in home between children with asthma and without asthma. No significant differences were found between included participants and those excluded from the analysis (Supplementary Table 1).

### The independent association between serum cotinine levels, BMI groups and asthma

After adjusting for age, gender, ethnicity, gender of the household reference person, and marital status of the household reference person, high-level serum cotinine was significantly associated with increased asthma risk compared with low-level serum cotinine [odds ratio (OR) = 1.190, 95% confidence interval (CI): 1.004–1.410 in Model 3], which was shown in Table 2. Children in Group B (OR = 1.275, 95%CI: 1.079–1.506) and Group C (OR = 1.636, 95%CI: 1.354–1.977) were significantly associated with increased asthma risk compared with children in Group A, adjusting for the above-mentioned covariates.

**Table 2:** The independent association between serum cotinine levels, BMI groups and asthma, and interaction between serum cotinine levels and BMI on asthma

	Model 1		Model 2		Model 3	
	OR (95%CI)	*P*	OR (95%CI)	*P*	OR (95%CI)	*P*
Serum cotinine^1^						
Low-level	Ref		Ref		Ref	
High-level	1.298 (1.104–1.527)	0.002	1.330 (1.129–1.565)	0.001	1.190 (1.004–1.410)	0.045
BMI groups						
Croup A	Ref		Ref		Ref	
Croup B	1.305 (1.110–1.535)	0.002	1.274 (1.081–1.503)	0.004	1.275 (1.079–1.506)	0.005
Croup C	1.731 (1.436–2.087)	< 0.001	1.663 (1.378–2.006)	< 0.0001	1.636 (1.354–1.977)	< 0.001
Interaction Low-level serum cotinine and Group A	Ref		Ref		Ref	
Low-level serum cotinine and Group B	1.360 (1.131–1.635)	0.001	1.330 (1.105–1.602)	0.003	1.329 (1.102–1.602)	0.003
Low-level serum cotinine and Group C	1.758 (1.440–2.145)	< 0.001	1.693 (1.389–2.063)	< 0.0001	1.670 (1.367–2.041)	< 0.001
High-level serum cotinine and Group A	1.332 (1.054–1.684)	0.017	1.371 (1.083–1.738)	0.009	1.199 (0.936–1.537)	0.150
High-level serum cotinine and Group B	1.549 (1.175–2.043)	0.002	1.539 (1.162–2.038)	0.003	1.359 (1.009–1.829)	0.043
High -level serum cotinine and Group C	2.125 (1.574–2.867)	< 0.001	2.065 (1.528–2.789)	< 0.0001	1.822 (1.357–2.448)	< 0.001
Interaction						
Attributable proportion of interaction (95%CI)	0.258 (0.083–0.433)		0.338 (0.234–0.443)		0.206 (0.075–0.337)	
Synergy index (95%CI)	1.934 (1.344–2.783)		2.225 (1.813–2.732)		1.617 (1.126–2.098)	

Ref, reference; *OR* Odds ratio, *CI* Confidence interval, *BMI* Body mass index, Group A: underweight and normal weight (< 85th percentile); Group B: overweight (≥ 85th percentile to < 95th percentile); and Group C: obesity (≥ 95th percentile)

^1^ low-level serum cotinine: < 0.0436 ng/ml and high-level serum cotinine: ≥ 0.0436 ng/ml

Model 1: unadjusted logistic model;

Model 2: adjusted for age and gender;

Model 3: adjusted for age, gender, ethnicity, gender of the household reference person, and marital status of the household reference person

### Interaction between serum cotinine levels and BMI on asthma

In Table 2, using low-level serum cotinine and Group A as the reference, there were significant interactions between low-level serum cotinine and Group B on asthma (OR = 1.329, 95%CI: 1.102–1.602), between low-level serum cotinine and Group C (OR = 1.670, 95%CI: 1.367–2.041) on asthma, between high-level serum cotinine and Group B (OR = 1.359, 95%CI: 1.009–1.829) on asthma, and between high-level serum cotinine and Group C on asthma (OR = 1.822, 95%CI: 1.357–2.448).

**Fig. 2:**
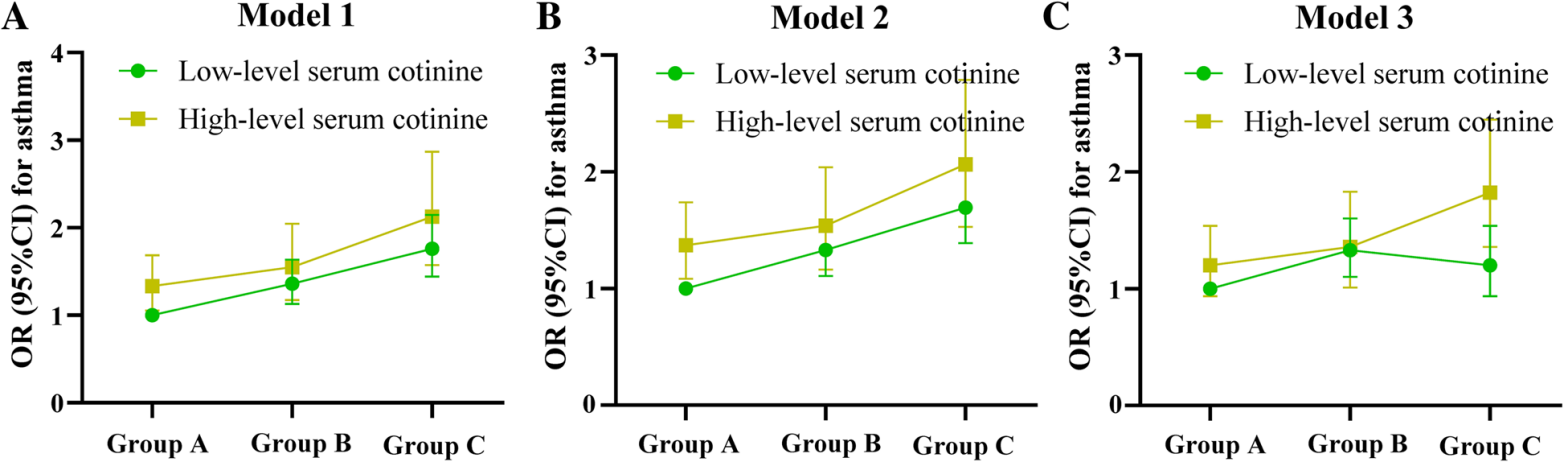
Interaction between serum cotinine [low-level (< 0.0436 ng/ml) and high-level (≥ 0.0436 ng/ml) serum cotinine] and BMI [Group A underweight and normal weight (< 85th percentile); Group B: overweight (≥ 85th percentile to < 95th percentile); and Group C: obesity (≥ 95th percentile) on childhood asthma, low-level serum cotinine and Group A was the reference. OR: odds ratio; CI: confidence interval; BMI: body mass index; Model 1: unadjusted logistic model (**A**); Model 2: adjusted for age and gender (**B**); Model 3: adjusted for age, gender, ethnicity, gender of the household reference person, and marital status of the household reference person (**C**)

The adjusted attributable proportion of interaction was 0.206 (95%CI: 0.075–0.337) and the adjusted synergy index was 1.617 (95%CI: 1.126–2.098) in Table 2, indicating that there was a significant synergistic effect of serum cotinine levels and BMI on asthma in Model 3. Fig. 2 provides a visual interaction between serum cotinine levels and BMI groups on asthma. For those with current asthma, the results of sensitivity analysis were shown in Supplementary Table 2, suggesting no significant synergistic effect of serum cotinine levels and BMI on asthma.

### Interaction between serum cotinine levels and BMI on asthma in different gender and ethnicity

In Table 3, the synergistic interaction between serum cotinine levels and BMI on asthma existed in both males [the adjusted attributable proportion of interaction = 0.173 (95%CI: 0.005–0.351) and the adjusted synergy index = 1.514 (95%CI: 1.050–2.184)] and females [the adjusted attributable proportion of interaction = 0.254 (95%CI: 0.061–0.447) and adjusted synergy index = 1.775 (95%CI: 1.222–2.579)]. In the population of non-Hispanic White and other Hispanic, there were synergistic interactions between serum cotinine levels and BMI on asthma with the adjusted attributable proportion of interaction = 0.317 (95%CI: 0.097–0.538), and the adjusted synergy index = 1.901 (95%CI: 1.272–2.840) in non-Hispanic White, and the adjusted attributable proportion of interaction = 0.423 (95%CI: 0.085–0.760), and the adjusted synergy index = 2.495 (95%CI: 1.241–5.013) in other Hispanic (Table 4).

**Table 3:** The interaction between serum cotinine levels and BMI on asthma in different gender

	Male		Female	
	OR (95%CI)	*P*	OR (95%CI)	*P*
Interaction^1^ Low-level serum cotinine and Group A	Ref		Ref	
Low-level serum cotinine and Group B	1.338 (1.01–1.771)	0.042	1.295 (0.942–1.780)	0.111
Low-level serum cotinine and Group C	1.541 (1.192–1.994)	0.001	1.893 (1.400–2.559)	< 0.001
High-level serum cotinine and Group A	1.184 (0.852–1.646)	0.311	1.236 (0.902–1.693)	0.186
High-level serum cotinine and Group B	1.360 (0.845–2.189)	0.203	1.390 (0.889–2.171)	0.147
High-level serum cotinine and Group C	1.832 (1.206–2.783)	0.005	1.814 (1.256–2.618)	0.002
Interaction				
Attributable proportion of interaction (95%CI)	0.173 (0.005–0.351)		0.254 (0.061–0.447)	
Synergy index (95%CI)	1.934 (1.344–2.783)		2.225 (1.813–2.732)	

Ref, reference; OR: odds ratio; *CI* Confidence interval, *BMI* Body mass index, Group A: underweight and normal weight (< 85th percentile); Group B: overweight (≥ 85th percentile to < 95th percentile); and Group C: obesity (≥ 95th percentile)

^1^ low-level serum cotinine: < 0.0436 ng/ml and high-level serum cotinine: ≥ 0.0436 ng/ml

The multivariate model adjusted for age, gender, ethnicity, gender of the household reference person, and marital status of the household reference person

**Table 4:** The interaction between serum cotinine levels and BMI on asthma in different ethnicity

	Other races		Mexican American		Non-Hispanic Black		Non-Hispanic White		Other Hispanic	
	OR (95%CI)	*P*	OR (95%CI)	*P*	OR (95%CI)	*P*	OR (95%CI)	*P*	OR (95%CI)	*P*
Interaction^1^ Low-level serum cotinine and Group A	Ref		Ref		Ref		Ref		Ref	
Low-level serum cotinine and Group B	1.067 (0.799–1.424)	0.657	1.646 (1.05–2.580)	0.030	1.401 (1.035–1.897)	0.029	1.222 (0.893–1.673)	0.208	1.185 (0.656–2.141)	0.570
Low-level serum cotinine and Group C	1.404 (1.063–1.856)	0.018	1.689 (1.123–2.540)	0.013	1.856 (1.290–2.669)	0.001	1.304 (0.974–1.746)	0.075	2.219 (1.464–3.363)	< 0.001
High-level serum cotinine and Group A	1.159 (0.746–1.800)	0.509	1.543 (0.872–2.730)	0.135	1.273 (0.873–1.858)	0.208	1.010 (0.784–1.301)	0.938	1.035 (0.611–1.755)	0.897
High-level serum cotinine and Group B	1.154 (0.506–2.629)	0.731	2.437 (0.997–5.958)	0.051	1.333 (0.855–2.077)	0.203	1.087 (0.752–1.571)	0.654	2.403 (0.826–6.993)	0.107
High -level serum cotinine and Group C	1.621 (0.953–2.756)	0.074	2.183 (1.110–4.294)	0.024	1.534 (0.899–2.616)	0.116	2.162 (1.66–2.815)	< 0.001	2.373 (1.164–4.834)	0.018
Interaction										
Attributable proportion of interaction (95%CI)	0.114 (-0.500–0.729)		0.240 (-0.107–0.587)		0.055 (-0.185–0.295)		0.317 (0.097–0.538)		0.423 (0.085–0.760)	
Synergy index (95%CI)	1.205 (0.440–3.300)		1.874 (0.830–4.231)		1.207 (0.599–2.431)		1.901 (1.272–2.840)		2.495 (1.241–5.013)	

Ref, reference; *OR* Odds ratio, *CI* Confidence interval, *BMI* Body mass index, Group A: underweight and normal weight (< 85th percentile); Group B: overweight (≥ 85th percentile to < 95th percentile); and Group C: obesity (≥ 95th percentile)

^1^ low-level serum cotinine: < 0.0436 ng/ml and high-level serum cotinine: ≥ 0.0436 ng/ml

The multivariate model adjusted for age, gender, ethnicity, gender of the household reference person, and marital status of the household reference person

## Discussion

Using interaction analysis, we found that there was a synergistic interaction between serum cotinine and overweight/obesity on asthma among a representative sample of the general population of American children. The presence of this interaction was found in male, female, Non-Hispanic White, and other Hispanic populations.

A study of six consecutive cycles of the 2003–2014 NHANES conducted by Zhang et al. found that the higher exposure to passive smoke reflected by serum cotinine was associated with higher odds of childhood asthma [10]. The results were consistent with ours that serum cotinine levels were significantly associated with asthma after being adjusted for age, gender, ethnicity, gender of the household reference person, and marital status of the household reference person. Another study on Iranian children under the age of 10 showed that passive smokers had higher cotinine levels than non-passive smokers, and cotinine was a predictive risk factor for asthma [11]. This research studied the cotinine levels in the serum, saliva, and urine of all patients participating in the study, while our study was limited to the data in the database and only studied the serum cotinine levels. These results suggested avoiding exposure to cigarette smoke during infancy and childhood may significantly improve the health of children [19]. Although smoking cessation programs exist for adults, including those parents of children with asthma, data from the NHANES found that more than one-third (35.4%) of non-smokers aged 3–17 were exposed to secondhand smoke [7]. It is possible to advocate in communities, schools, medical institutions and other places to protect young people from the dangers of passive smoking by implementing smoking bans, and educating family caregivers and young people on maintaining a healthy weight.

The prevalence of asthma and overweight in children has increased simultaneously in recent decades [20]. Some studies suggested that being overweight or obese in children was a risk factor for asthma [21, 22]. Egan et al. [21] examined six cohort studies and reported that children who were overweight or obese had a 50% increased risk of physician-diagnosed asthma compared with children of normal weight. These results are supported by a meta-analysis by Chen et al. [22], which demonstrated that the risk of asthma in obese children is proportional to the BMI value. These studies are consistent with our findings that both overweight and obesity were associated with childhood asthma. However, Lang et al. [23] showed that asthma could be driven by both duration and severity of overweight, and a meta-analysis [24] concluded that there was a bidirectional association between obesity and asthma in childhood and adolescence. The relationship between asthma and overweight/ obesity remains controversial, and the underlying causal relationship is unclear [21, 25], and further researches are needed [26].

To our knowledge, this is the first study to explore the interaction between serum cotinine and BMI on asthma in US general children. Although the reason for the interaction remains unclear, their effect on the airways could explain this effect. Obese subjects have been reported to exhibit significant increases in bronchial hyper-responsiveness over healthy subjects [27]. Similar to obesity, smoking may also directly induce hyper-responsiveness of the airways, resulting in increased asthmatic symptoms [12]. A similar explanation that may be possible was that the airway hyperresponsiveness (AHR) pathway is involved in obesity and it is suggested to play a broad role in obesity and associated complications. Future studies should improve our understanding of the different mechanisms and pathways that underlie obese asthma.

Based on our findings, the interaction between serum cotinine and BMI on asthma existed in US general children with a history of asthma, whereas it was not found in children with current asthma. The reason may be limited by the sample size of children with current asthma (6.87 weighted %), when the co-occurrence of the two risk combinations (high-level serum cotinine and overweight/obesity) is not significant for children with current asthma. Further studies are needed to analyze the factors associated with children with current asthma to suggest relevant interventions.

In subgroup analyses, there was synergistic interactions between serum cotinine and BMI on asthma in both male and female populations. Both high and low serum cotinine levels in combination with obesity were associated with an increased risk of childhood asthma, suggesting that clinicians should pay special attention to weight interventions in the population. For the population of non-Hispanic White and other Hispanic, attention should be paid to both high-level passive smoke exposure and weight interventions.

The strengths of this study were as follows. Firstly, there were few studies on the relationship between serum cotinine, BMI and childhood asthma. Our research provided the basis for showing that there was a synergistic interaction between serum cotinine and overweight/obesity on asthma. Second, the study population was relatively large with a sufficient number of children in the U.S. Third, cotinine is a biomarker of nicotine exposure and cotinine level is a marker of the passive smoker, which can avoid biases related to self-reports.

A few limitations were in our study. First, NHANES data are cross-sectional, and causal or longitudinal relationships cannot be determined. Second, we adjusted the widely-used confounders, and did not adjust for other confounders due to retrospective and database information, such as allergies, gene-related covariates, etc. that may be related to childhood asthma [28]. Third, cotinine could be measured in the blood, saliva, hair or nails of contacts. Urine and serum testing methods are widely used and are considered reliable [29]. In this study, we only evaluated the cotinine in the blood, and we did not evaluate the cotinine content in the urine, which was limited to the database. Third, we could not determine the level of prenatal cotinine because cotinine reflects the level of nicotine exposure. Nicotine may damage the development of the airway in the uterus, resulting in decreased lung function and increased childhood respiratory-related diseases [30]. Further studies were needed to measure cotinine levels from more dimensions considering saliva, and urine, characterize asthma beyond ever been told you have asthma, consider other anthropometric indices and biomarkers, and conduct prospective studies on BMI, cotinine levels and childhood asthma to provide more evidence.

## Conclusion

In this study, we found a synergistic interaction between serum cotinine and overweight/obesity on asthma among a representative sample of the general population of American children. For children with asthma, although both being overweight or obese or passive smoking are known to be harmful, both intensive weight interventions in overweight or obese children and intensive passive smoking interventions in children exposed to the environment may be important.

## Supplementary Information

**Additional file 1:** **Supplementary Table 1.** Comparison between included participants and those excluded from the analysis. **Supplementary Table 2.** Sensitivity analysis for current asthma^1^.

## Data Availability

The datasets generated and/or analysed during the current study are available in the NHANES database, https://www.cdc.gov/nchs/nhanes/.

## Abbreviations

NHANES: National health and nutrition examination surveys
PIR: Ratio of family income to poverty
NCHS: National center for health statistics
ID HPLC-APCI MS/MS: Sotope dilution–high-performance liquid chromatography / atmospheric pressure chemical ionization tandem mass spectrometry
BMI: Body mass index
SE: Standard error
OR: Odds ratio
CI: Confidence interval
AHR: Airway hyperresponsiveness
