# Supplementary material for: Interaction between serum cotinine and body mass index on asthma in the children: a cross-sectional study
Source: BMC Pediatr. 2022 Aug 23;22:499. doi: 10.1186/s12887-022-03571-0 (PMC9400283; doi:10.1186/s12887-022-03571-0)
Supplement: Supplementary file 1 — Additional file 1: Supplementary Table 1. Comparison between included participants and those excluded from the analysis. Supplementary Table 2. Sensitivity analysis for current asthma1. [file 12887_2022_3571_MOESM1_ESM.docx]

Supplementary Table 1. Comparison between included participants and those excluded from the analysis

| Characteristic | Total  (n=27,921) | Groups | | Statistic^1^ | *P* |
| --- | --- | --- | --- | --- | --- |
|  |  | Included participants  (n=12,967) | Included and excluded participants  (n=14,954) |  |  |
| Age, years, Mean ± SD | 7.86 ± 2.84 | 7.87 ± 2.84 | 7.86 ± 2.85 | t=0.340 | 0.733 |
| PIR, M (Q1, Q3) | 1.41 (0.76,2.81) | 1.42 (0.76,2.82) | 1.41 (0.76,2.80) | Z=0.737 | 0.461 |
| Serum cotinine, ng/ml, M (Q1, Q3) | 0.05 (0.02,0.31) | 0.05 (0.02,0.31) | 0.05 (0.02,0.31) | Z=-0.597 | 0.551 |
| Gender, n (%)^3^ |  |  |  | χ^2^=0.010 | 0.920 |
| Male | 14,173 (50.76) | 6,578 (50.73) | 7,595 (50.79) |  |  |
| Female | 13,748 (49.24) | 6,389 (49.27) | 7,359 (49.21) |  |  |
| Ethnicity, n (%)^3^ |  |  |  | χ^2^=9.086 | 0.059 |
| Mexican American | 7,767 (27.82) | 3,537 (27.28) | 4,220 (28.22) |  |  |
| Other Hispanic | 2,395 (8.58) | 1,087 (8.38) | 1,308 (8.75) |  |  |
| Non-Hispanic White | 7,677 (27.50) | 3,674 (28.33) | 4,013 (26.84) |  |  |
| Non-Hispanic Black | 7,496 (26.85) | 3,466 (26.73) | 4,030 (26.95) |  |  |
| Other races | 2,586 (9.26) | 1,203 (9.28) | 1,383 (9.25) |  |  |
| BMI groups, n (%)^3^ |  |  |  | χ^2^=0.312 | 0.855 |
| Croup A | 18,050 (64.65) | 8,405 (64.82) | 9,645 (64.50) |  |  |
| Croup B | 4,448 (15.93) | 2,056 (15.86) | 2,392 (16.00) |  |  |
| Croup C | 5,423 (19.42) | 2,506 (19.33) | 2,917 (19.51) |  |  |
| Gender of the household reference person^2^, n (%)^3^ |  |  |  | χ^2^=1.393 | 0.238 |
| Female | 15,120 (54.16) | 7,072 (54.54) | 8,048 (53.83) |  |  |
| Male | 12,797 (45.84) | 5,895 (45.46) | 6,902 (46.17) |  |  |
| Age of the household reference person, years, M (Q1, Q3) | 36.00 (30.00, 42.00) | 36.00 (30.00, 42.00) | 36.00 (30.00, 42.00) | Z=0.537 | 0.591 |
| Education of the household reference person, n (%)^3^ |  |  |  | χ^2^=5.624 | 0.229 |
| Less than 9th grade | 3,495 (12.74) | 1,624 (12.52) | 1,871 (12.94) |  |  |
| 9-11th grade | 6,148 (22.41) | 2,849 (21.97) | 3,299 (22.81) |  |  |
| High school graduate | 6,510 (23.73) | 3,079 (23.74) | 3,431 (23.72) |  |  |
| Some college or AA degree | 6,866 (25.03) | 3,291 (25.38) | 3,575 (24.72) |  |  |
| College graduate or higher | 4,411 (16.08) | 2,124 (16.38) | 2,287 (15.81) |  |  |
| Marital status of the household reference person, n (%)^3^ |  |  |  | χ^2^=1.751 | 0.882 |
| Divorced | 16,985 (62.41) | 8,088 (62.37) | 8,897 (62.44) |  |  |
| Living with partner | 848 (3.12) | 387 (2.98) | 461 (3.24) |  |  |
| Married | 2,600 (9.55) | 1,252 (9.66) | 1,348 (9.46) |  |  |
| Never married | 1,473 (5.41) | 706 (5.44) | 767 (5.38) |  |  |
| Separated | 3,456 (12.70) | 1,646 (12.69) | 1,810 (12.70) |  |  |
| Widowed | 1,853 (6.81) | 888 (6.85) | 965 (6.77) |  |  |
| Anyone smokes in home, n (%)^3^ |  |  |  | χ^2^=0.115 | 0.735 |
| No | 21,939 (79.02) | 10,235 (78.93) | 11,704 (79.10) |  |  |
| Yes | 5,825 (20.98) | 2,732 (21.07) | 3,093 (20.90) |  |  |
| Asthma, n (%)^3^ |  |  |  | χ^2^=0.009 | 0.926 |
| No | 23,457 (84.01) | 10,891 (83.99) | 12,566 (84.03) |  |  |
| Yes | 4,464 (15.99) | 2,076 (16.01) | 2,388 (15.97) |  |  |

PIR, ratio of family income to poverty; BMI, body mass index; SD, standard deviation; M (Q1, Q3), the median and interquartile range; Group A: underweight and normal weight (<85th percentile); Group B: overweight (≥85th percentile to <95th percentile); and Group C: obesity (≥95th percentile).

^1.^ t, t-test; Z, the Mann-Whitney U rank-sum test; χ^2^, the chi-square test.

^2.^ The household reference person is the first household member, 18 years of age or older who is listed on the Screener household member roster who owns or rents the residence where members of the household reside.

^3.^ weighted %.

Supplementary Table 2. Sensitivity analysis for current asthma^1^

|  | Model 1 | | Model 2 | | Model 3 | |
| --- | --- | --- | --- | --- | --- | --- |
|  | OR (95%CI) | *P* | OR (95%CI) | *P* | OR (95%CI) | *P* |
| Serum cotinine^2^ |  |  |  |  |  |  |
| Low-level | Ref |  | Ref |  | Ref |  |
| High-level | 1.098 (0.907-1.33) | 0.336 | 1.112 (0.917-1.348) | 0.278 | 0.975 (0.789-1.204) | 0.811 |
| BMI groups |  |  |  |  |  |  |
| Croup A | Ref |  | Ref |  | Ref |  |
| Croup B | 0.968 (0.729-1.284) | 0.819 | 0.954 (0.718-1.267) | 0.743 | 0.965 (0.726-1.283) | 0.804 |
| Croup C | 1.566 (1.251-1.960) | 0.000 | 1.524 (1.220-1.902) | 0.000 | 1.510 (1.209-1.886) | 0.000 |
| Interaction  Low-level serum cotinine and Group A | Ref |  | Ref |  | Ref |  |
| Low-level serum cotinine and Group B | 0.942 (0.687-1.290) | 0.707 | 0.931 (0.677-1.279) | 0.656 | 0.943 (0.685-1.299) | 0.719 |
| Low-level serum cotinine and Group C | 1.656 (1.256-2.183) | 0.000 | 1.613 (1.229-2.118) | 0.001 | 1.613 (1.225-2.125) | 0.001 |
| High-level serum cotinine and Group A | 1.117 (0.869-1.436) | 0.384 | 1.136 (0.883-1.462) | 0.320 | 0.969 (0.737-1.274) | 0.820 |
| High-level serum cotinine and Group B | 1.159 (0.700-1.918) | 0.565 | 1.148 (0.693-1.900) | 0.591 | 0.998 (0.594-1.675) | 0.993 |
| High -level serum cotinine and Group C | 1.495 (1.103-2.025) | 0.010 | 1.464 (1.080-1.986) | 0.015 | 0.943 (0.685-1.299) | 0.719 |
| Interaction |  |  |  |  |  |  |
| Attributable proportion of interaction | 0.260 (0.084-0.436) |  | 0.258 (0.083-0.434) |  | 0.111 (-0.110-0.332) |  |
| Synergy index (95%CI) | 1.913 (1.337-2.738) |  | 1.934 (1.344-2.783) |  | 1.316 (0.809-2.142) |  |

^1.^ The positive answer to the question “During the past 12 months, have you had an episode of asthma or an asthma attack?”

^2.^ low-level serum cotinine: <0.0436 ng/ml and high-level serum cotinine: ≥0.0436 ng/ml.

Ref, reference; OR: odds ratio; CI: confidence interval; BMI, body mass index; Group A: underweight and normal weight (<85th percentile); Group B: overweight (≥85th percentile to <95th percentile); and Group C: obesity (≥95th percentile).

Model 1: unadjusted logistic model;

Model 2: adjusted for age and gender;

Model 3: adjusted for age, gender, ethnicity, gender of the household reference person, and marital status of the household reference person.
